# Learning to Combine Local and Global Image Information for Contactless Palmprint Recognition

**DOI:** 10.3390/s22010073

**Published:** 2021-12-23

**Authors:** Marjan Stoimchev, Marija Ivanovska, Vitomir Štruc

**Affiliations:** 1 Institut Jožef Stefan, Jamova Cesta 39, 1000 Ljubljana, Slovenia; 2 Faculty of Electrical Engineering, University of Ljubljana, Tržaška Cesta 25, 1000 Ljubljana, Slovenia; marija.ivanovska@fe.uni-lj.si (M.I.); vitomir.struc@fe.uni-lj.si (V.Š.)

**Keywords:** palmprint recognition, contactless palmprint images, elastic deformations, convolutional neural networks, deep learning, ArcFace loss, center loss, discriminative feature learning

## Abstract

In the past few years, there has been a leap from traditional palmprint recognition methodologies, which use handcrafted features, to deep-learning approaches that are able to automatically learn feature representations from the input data. However, the information that is extracted from such deep-learning models typically corresponds to the global image appearance, where only the most discriminative cues from the input image are considered. This characteristic is especially problematic when data is acquired in unconstrained settings, as in the case of contactless palmprint recognition systems, where visual artifacts caused by elastic deformations of the palmar surface are typically present in spatially local parts of the captured images. In this study we address the problem of elastic deformations by introducing a new approach to *contactless palmprint recognition* based on a novel CNN model, designed as a two-path architecture, where one path processes the input in a holistic manner, while the second path extracts local information from smaller image patches sampled from the input image. As elastic deformations can be assumed to most significantly affect the global appearance, while having a lesser impact on spatially local image areas, the local processing path addresses the issues related to elastic deformations thereby supplementing the information from the global processing path. The model is trained with a learning objective that combines the Additive Angular Margin (ArcFace) Loss and the well-known center loss. By using the proposed model design, the discriminative power of the learned image representation is significantly enhanced compared to standard holistic models, which, as we show in the experimental section, leads to state-of-the-art performance for contactless palmprint recognition. Our approach is tested on two publicly available contactless palmprint datasets—namely, IITD and CASIA—and is demonstrated to perform favorably against state-of-the-art methods from the literature. The source code for the proposed model is made publicly available.

## 1. Introduction

**Figure 1:**
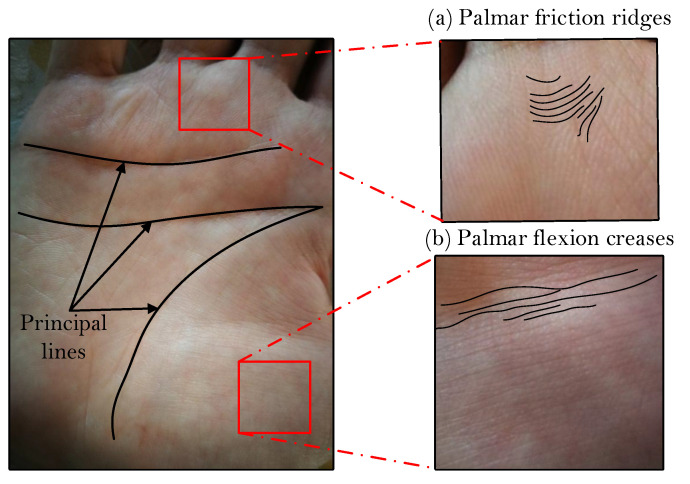
Illustration of the main palmprint characteristics/features commonly used for identity inference in palmprint recognition systems: (**a**) palmar friction ridges and (**b**) palmar flexion creases.

The area of palmprint recognition is a well-established subfield of biometrics and has been widely deployed commercially throughout the years [1,2]. Palmprint recognition techniques typically rely on two kinds of features for identity recognition in addition to the obvious principle lines, i.e., palmar friction ridges (the ridge and valley structures like fingerprint) and the palmar flexion creases (discontinuities in the epidermal ridge patterns), as illustrated in Figure 1a,b, respectively [1,3,4]. These characteristics represent rich sources of discriminative information based on which efficient recognition models can be designed.

Palmprint-based biometric systems have been increasingly researched recently due to their high recognition accuracy, usability, and acceptability [1,2].

**Figure 2:**
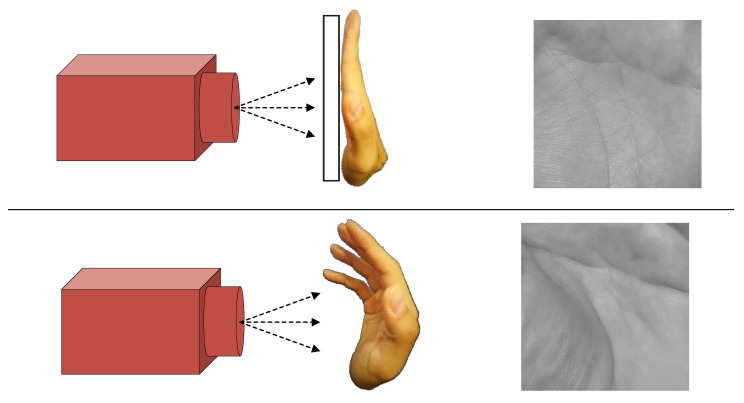
Illustrative comparison between a contact-based (at the top) and a contactless acquisition procedure used in palmprint recognition systems. Because the placement of the hand is not constrained by a contact surface, images acquired in a contactless manner exhibit larger variability (also due to elastic deformations of the palmar surface) as shown by the example palmprint images on the right. Note that both example palmprints originate from the same subject.

Over the last few years, there has been a transition from contact-based to contactless palmprint recognition approaches with a considerable amount of research focusing on the latter problem [1,2,4,5,6,7,8,9,10,11]. Different from palmprint images captured with contact-based sensors, palmprint images captured in a contactless manner may be taken with commercial off-the-shelf cameras and in unconstrained environments [1]. This not only widens the range of potential application scenarios where palmprint recognition can be used, but also brings improved convenience to users. Additionally, contactless palmprint acquisition offers many other benefits over contact-based alternatives, such as higher user friendliness, increased privacy, and better hygiene [4]. Nevertheless, capturing palmprints in a contactless manner also leads to new challenges related to increased appearance variability caused by rotations, ambient illumination, translations, different distances from the sensor, elastic deformations, as well as by the presence of higher levels of noise [12]. Among these, elastic deformations of the palmar surface (originating from the lack of contact surface that would constrain the placement of the hand) are one of the biggest issues for the recognition technology. This is illustrated in Figure 2, where a contact-based (shown at the top) as well as a contactless (shown at the bottom) acquisition procedures are presented. As can be seen, the unconstrained placement of the hand typically results in palmprint samples that exhibit significant deformations compared to images captured with contact-based sensors. It has been shown that conventional palmprint recognition methods often degrade in performance when applied on such types of data due to the severe inter-class variability induced by the acquisition procedure. To avoid such performance degradations, more powerful models capable of extracting robust and discriminative images representations are needed.

To address the problems encountered in challenging deployment scenarios, researchers are increasingly looking into deep-learning (DL) techniques when designing biometric solutions. Such techniques are capable of: (i) extracting knowledge and learning discriminative representations from (partially noisy) data automatically and in an end-to-end manner, (ii) adapting to the characteristics of the biometric samples, and (iii) achieving more accurate recognition performance in less-constrained environments compared with traditional hand-crafted feature extraction techniques. Several recent solutions, therefore, consider DL techniques for contactless palmprint recognition [1,4,6,13,14].

However, they exhibit at least one (or more) of the following shortcomings. First, the majority of existing techniques process input images in a holistic manner, where the local information that is preserved in the computed representation is limited only to the most discriminative parts of the input. In unconstrained settings this can be problematic, since different parts of the input might be elastically deformed, which greatly affects the performance of such methods. Second, deep-learning models are often designed for closed-set recognition tasks (using a softmax over a fixed set of classes), raising questions about the discriminability of the feature representations learned internally for open-set problems.

Moreover, existing (hybrid) methods that utilize both global and local image information fail to do so in a transparent way, that would allow interpretation of the results and lead to explainable decisions. This has implications for the future application of such models and their compliance with existing privacy laws, such as GDPR [15,16].

In this work, we aim to address the problems outlined above by presenting a hybrid DL based technique for contactless palmprint recognition, which: (i) utilizes both global and local information, (ii) learns highly discriminative features with considerable generalization capability, while not requiring extensive parameter tuning, and (iii) ensures interpretable recognition results. To the best of our knowledge, the proposed approach represents the first hybrid DL technique developed specifically for contactless palmprint recognition. Different from other DL based solutions, our model also exhibits a high-level of robustness to elastically deformed images and, as we show in the experimental section, ensures state-of-the-art recognition performance.

In summary, we make the following contributions in this study:

- We present a hybrid DL approach for contactless palmprint recognition based on a novel two-path network architecture that takes global as well as local image information into account.
- We propose an attention-based channel pooling operation, which is capable of (adaptively) extracting the most discriminative (local) information from sampled patches of the given input image.
- We introduce thin plate splines (TPS) as an augmentation procedure for explicitly modeling elastic deformations and demonstrate that the proposed augmentation procedure is beneficial for recognition performance.
- We show the benefit of combining local and global image information into discriminative representations that lead to a state-of-the-art performance for contactless palmprint recognition on two publicly available datasets.

## 2. Related Work

In this section we discuss prior work related to this study and cover some of the most important representatives of conventional as well as deep learning-based palmprint recognition methods. The reader is referred to some of the excellent surveys on palmprint recognition for a more in-depth coverage of the field [17,18,19].

### 2.1. Conventional Methods

Early work on palmprint recognition focused on hand-crafted feature extraction techniques to encode the palmprint structure. Jia et al. [8], for example, presented a palmprint descriptor, called Histogram of Oriented Lines (HOL) inspired by the established Histogram of Oriented Gradients (HOG) descriptor [8] and developed a recognition approach around HOL that also considered subspace learning methods for dimensionality reduction, i.e., PCA, LDA, and SRDA, as well as their corresponding kernel versions KPCA, KLDA, and KSRDA. The proposed approach was tested on two palmprint datasets, i.e., PolyU II [20] and PolyU MB [21]. Kumar [2] explored the use of improved alignment and matching strategies for contactless palmprint recognition using several publicly available contactless palmprint datasets and showed that these lead to state-of-the-art performance. Wu et al. [22] presented a contactless palmprint verification method that used an isotropic filter for preprocessing and SIFT based feature extraction and matching. The proposed method utilized a two-stage strategy, i.e., in the first stage, an iterative RANSAC (I-RANSAC) algorithm was employed to remove the mismatched points, and in the second stage, Local Palmprint Descriptors (LPDs) were extracted at SIFT keypoints and used to further refine the keypoint matching procedure. As a detection score, the authors used the final number of matched SIFT keypoints.To evaluate the performance of the proposed approach, two publicly available contactless palmprint databases were considered, i.e., the IIT Delhi (IITD) Touchless Palmprint Database [23], and the CASIA palmprint database [24]. Zheng et al. [11] presented a new descriptor referred to as Difference of Vertex Normal Vectors (DoN) for 2D palmprint matching. The descriptor is based on the ordinal measure which partially describes the difference of the neighboring points’ normal vectors. Using the DoN descriptor, the authors were able to extract 3D information from 2D palmprint images, which was demonstrated to be highly stable under commonly occurring illumination variations during contactless imaging. The proposed method was evaluated on several publicly available 2D palmprint databases in identification as well as verification mode with highly encouraging results.

While the solutions reviewed above achieved impressive performance for palmprint recognition, they are still limited in the amount of discriminative information they are able to extract from the input images given their hand-crafted design. A richer and more descriptive set of features can in general be obtained through the use of deep learning models, such as the one presented in this work.

### 2.2. Deep Learning Methods

More recently, the focus in the field of palmprint recognition shifted from traditional feature engineering approaches to the deep learning based solutions [1,13,14,25]. Fei et al. [18], for example, evaluated a number of feature extraction methods for contactless palmprint recognition, including four deep learning architectures, i.e., AlexNet [26], VGG-16 [27], GoogLeNet [28] and Res-Net-50 [29]. These networks were pre-trained on ImageNet [30] and then fine tuned using extracted palmprint regions of interest (ROIs). The authors concluded that deep learning methods achieve comparable or even higher performance than conventional palmprint methods. Genovese et al. [6] introduced the PalmNet architecture, a novel Convolutional Neural Network (CNN) that is capable of fine tuning specific palmprint filters through an unsupervised procedure based on Gabor responses and Principal Component Analysis (PCA), while not requiring class labels during the training process. The authors validated their approach on several publicly available datasets, i.e., the CASIA palmprint database V1 [24], the IITD database (Version 1.0) [23], the REgim Sfax Tunisia (REST) hand database 2016 [25] and the Tongji Contactless Palmprint Dataset [5]. In all cases, they obtained recognition accuracies greater than those of the prior methods from the literature [6]. Svoboda et al. [1], presented a Siamese-type CNN for palmprint image recognition based on a novel loss function related to the *d*-prime index. Their approach automatically learns features from the data, thus not requiring extensive parameter tuning. By using their design, the authors achieved greater class separation between the genuine and the impostor score distributions and better scalability than competing methods, while not requiring large amounts of training data. The authors also achieved state-of-the-art verification results on the standard IIT Delhi [23] and CASIA palmprint [24] datasets. Liu and Kumar [4], designed a fully convolutional and highly optimized network that used a soft-shifted triplet (SSTL) loss function to learn discriminative palmprint features. The presented method was shown to offer superior generalization capability over different publicly available contactless palmprint databases, while not requiring database-specific parameter tuning. By using the proposed design, the authors consistently outperformed several classical and state-of-the-art palmprint recognition methods in identification, as well as in verification experiments. Zhu et al. [31] introduced an adversarial metric learning methodology for palmprint recognition and reported superior performance over competing techniques on eight different datasets.

The approach in this paper is related to the above methods in that it also uses deep learning to address the problem of contactless palmprint recognition but differently from the existing solutions, it takes not only global image appearance into account, but also the most discriminative local image cues that are aggregated through a novel attention based channel pooling operation. Moreover, to further boost the verification performance the proposed model uses a powerful learning objective that allows it to learn highly discriminative feature representations.

## 3. Proposed Method

In this section we present our new DL-based methodology for contactless palmprint recognition. First, we explain how the model encodes global and local image characteristics from the palmprint images. Next, we introduce the attention mechanism. Finally, we proceed to the learning mechanism used to further enhance the discriminative power and the quality of the learned deep palmprint features.

### 3.1. Overview of the Proposed Model

**Figure 3:**
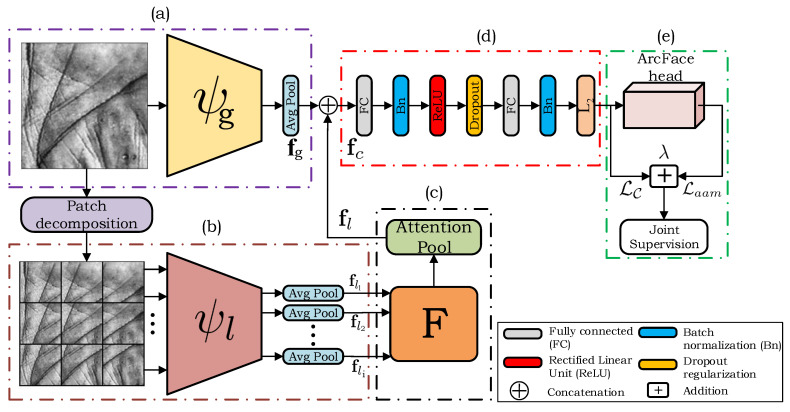
Overview of the proposed method for contactless palmprint recognition. Our model is designed as a Two-path architecture. The first path of the model, marked (**a**), extracts holistic image information through a backbone CNN feature extractor. The second path, marked (**b**), represents the local processing path where the sampled patches from a fixed grid are processed through a parallel Siamese-based CNN feature extractor with shared model parameters. The next part of the model, marked (**c**), extracts the most relevant local information from the local image patches with a new channel-wise attention pooling mechanism. The global and the aggregated local features are then combined in (**d**). The final part of the model, marked (**e**), is only used during training and represents the joint supervision with the ArcFace and the center losses.

The idea behind our approach is illustrated on Figure 3. Compared to other relevant deep learning methods, our method relies on a *dual-path model architecture*, where one path processes the input in a holistic manner through a backbone CNN feature extractor and the second path captures local (patch-based) information through a backbone Siamese-based CNN feature extractor. In the local processing path feature representations are extracted from each of the patches sampled from the input image and aggregated by a novel pooling mechanism, called *channel-wise attention pooling*. At the final stage, the global holistic and the aggregated local features are combined by using a simple concatenation procedure. The model is trained end-to-end with a learning objective that combines the Additive Angular Margin (ArcFace) loss with the center loss. This overall training objective helps the model learn highly discriminative features, which is crucial for contactless palmprint recognition. The overall idea with our design is that the complementary information is learned within the two processing paths, where the local path compensates for the image characteristics that are not captured well by the global processing path. Since the presence of elastic deformations of the palmar surface is a major obstacle for palmprint images acquired in a contactless manner, we designed our model under the assumption that a combination of such global and local information and joint supervision through the combined loss, would lead to more robust and discriminative image representations. As we show in the experimental section, the addition of local image representations contributes toward better and more robust overall recognition performance. The following sections provide detailed explanations of all the building blocks of the proposed method.

### 3.2. The Global Processing Path

**Figure 4:**
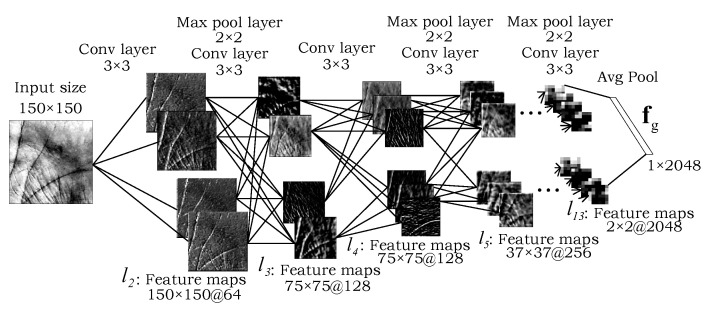
Detailed illustration of the global processing path from Figure 3a, along with the feature maps extracted at different levels of the model. A fully convolutional VGG-16 backbone is used to extract a global palmprint representation $f_{g}$ from the input image.

The global processing part of the model (shown in Figure 3a and Figure 4) is responsible for capturing global palmprint characteristics, similarly to standard CNNs used for recognition purposes in other areas of biometrics [27,32,33]. The processing path consists of a simple CNN backbone feature extractor $\psi_{g}$ that takes a palmprint ROI $x\in R^{w\times h\times 3}$ as input, where *w* and *h* denote image dimensions, and generates a global image representation $f_{g}$ as the results. Formally, this can be written as:

(1) $$f_{g}=\psi_{g}\left(x,\theta_{g}\right)\in R^{d},$$

where $\theta_{g}$ denotes the parameters of the global processing path that need to be learned during training. While any backbone CNN can be used in our approach, we select the popular VGG-16 model [27] in this work because of (i) its competitive performance, and (ii) the fact that an open source implementation is publicly available.

To make the global processing path applicable to differently sized input images, we make sure the backbone CNN is fully convolutional. Thus, the *d*-dimensional global features $f_{g}\in R^{d}$ are generated through an average pooling layer in case of the VGG-16 backbone, as also illustrated in Figure 4.

### 3.3. The Local Patch-Based Processing Path

**Figure 5:**
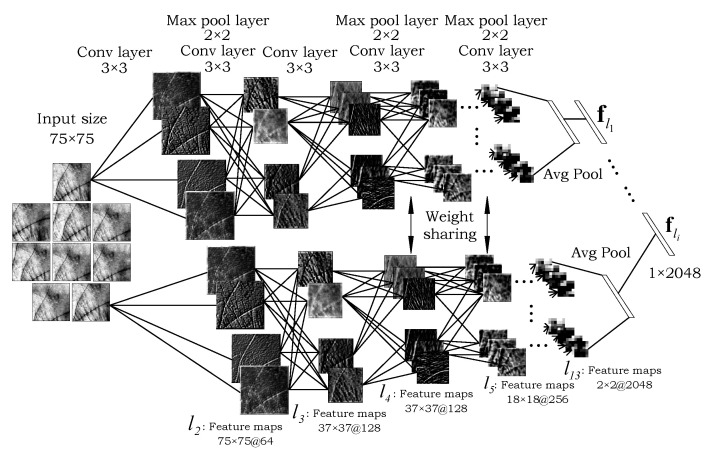
Detailed illustration of the local processing path from Figure 3b, along with the feature maps learned at different levels of the model. Image patches, sampled from the input image in a grid-like manner, are processed by a parallel backbone Siamese CNN with shared model parameters resulting in *N* local image features $f_{l_{i}}$, where i=1,…,N.

In the second processing path of our model (shown in Figure 3b and in detail in Figure 5), the global palmprint image is first decomposed into *N* smaller patches—sampled from a fixed grid with overlap (Figure 5). Here, *N* is an open hyper-parameter that can be set depending on the data characteristics. This processing path differs from the global path in that it represents a Siamese CNN with shared model parameters. Thus, the same feature extractor is used for all image patches. This design is able to process all *N* patches in a single shot. By focusing only on a small palmprint area at the time, the local processing path is able to encode important local image characteristics that are not properly represented in the holistic representation extracted by the global processing path. If we denote the set of sampled local patches as ${\left\{x_{i}\right\}}_{i=1}^{N}$, where $x_{i}\in R^{w^{\prime }\times h^{\prime }\times 3}$ and $w^{\prime }\leq w$ and $h^{\prime }\leq h$, then the local image representations can be described as:

(2) $$f_{l_{i}}=\psi_{l_{i}}\left(x_{i},\theta_{l}\right)\in R^{d},$$

where $\psi_{l_{i}}$ denotes the *i*-th component of the Siamese structure, $\theta_{l}$ are the shared model parameters, $f_{l_{i}}={\left[f_{i1},f_{i2},\ldots ,f_{id}\right]}^{T}$ is the *i*-th *d*-dimensional local feature vector and i=1,…,N.

Similarly as in the global processing path, the local processing path can be implemented with arbitrary backbone CNNs in the Siamese structure. However, we again utilize a fully convolutional VGG-16 model in this work to make our approach more coherent as illustrated in Figure 5. Next, we aggregate all local feature vectors into a N×d dimensional feature matrix F as shown in Equation (3), where *N* denotes the number of patches and *d* is the feature dimension, i.e.,

(3) $$F=\left[\begin{array}{cccc} f_{11} & f_{12} & \cdots & f_{1d}\\ f_{21} & f_{22} & \cdots & f_{2d}\\ \vdots & \vdots & \ddots & \vdots \\ f_{N1} & f_{N2} & \cdots & f_{Nd} \end{array}\right]\in R^{N\times d}$$

The generated feature matrix then serves as the basis for a novel pooling operation that is described in depth in the next section.

### 3.4. The Attention Mechanism

**Figure 6:**
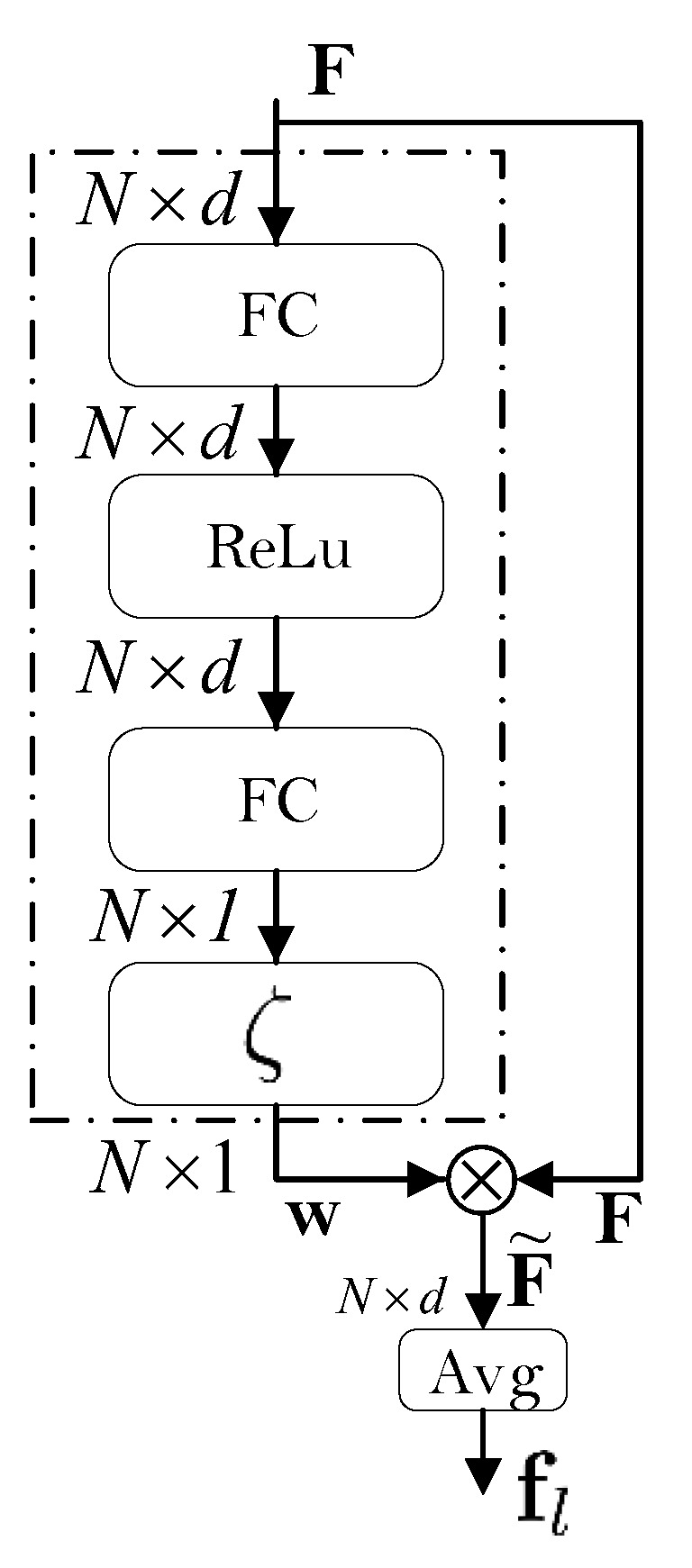
Illustration of the channel-wise attention-pooling mechanism. The local feature matrix F is reweighted along the patch dimension to give more importance to more informative local image patches. Shown is the architecture, implemented in the model we have used.

In order to obtain the final most relevant *d*-dimensional feature representation from the local feature matrix F, a novel pooling operation called *channel-wise attention-pooling* is proposed in this paper. This operation is implemented through a dedicated CNN, as shown in Figure 6, and has its own trainable parameters. Suppose the output of the local processing path is the N×d feature matrix F, then the output from the attention-pooling layer can be calculated as follows:

(4) $$\tilde{F}=F⊙\left(w\cdot 1_{d}^{T}\right)\in R^{N\times d},$$

where

(5) $$w=\zeta \left(y\right)\in R^{N},$$

and where $\tilde{F}$ denotes the weighted feature matrix, ⊙ represent the element-wise or Hadamard product, w is a vector of weights, $1_{d}\in R^{d}$ is a *d*-dimensional vector of all ones, y is the output from the last fully connected (FC) layer with the number of output units equal to the number of patches *N*, and ζ is the softmax activation function. After applying the softmax activation function, the vector of weights $w={\left[w_{1},w_{2},\ldots ,w_{N}\right]}^{T}$ has the following property:

(6) $$\sum_{i=1}^{N}w_{i}=1,$$

and can be considered as a vector of probabilities. The final local feature vector $f_{l}$ is obtained by averaging the weighted feature matrix over the patch dimension (indicated by the superscript) as shown in Equation (7), i.e.,

(7) $$f_{l}=\frac{1}{N}\sum_{i=1}^{N}{\tilde{F}}^{\left(i\right)}\in R^{d},$$

The main idea behind the proposed channel-wise attention-pooling mechanism is to encourage the model to focus on the most discriminative parts of the input, while giving less importance to less discriminative parts. The proposed approach also offers a straight-forward way of exploring the importance of each image part (by examining the weights in w) which contributes towards high explainability of our model. In summary the mapping $\psi_{a}$ performed by the proposed attention mechanism is formally described as:

(8) $$\tilde{F}=\psi_{a}\left(F,\theta_{a}\right),$$

where $\theta_{a}$ stands for the set of parameters of the attention block from Figure 6.

### 3.5. Combining Information

In the next step, the global and local features are concatenated to form the combined feature representation $f_{c}$, i.e.,

(9) $$f_{c}=f_{g}⊕f_{l}\in R^{2d},$$

where ⊕ is the concatenation operator. This representation is then fed to a series of additional processing layers with parameters $\theta_{f}$, as shown in Figure 3d. Various forms of regularizations are also included to prevent overfitting and to enhance the stability of the model during the training process. One such form of regularization is the dropout regularization, which prevents the model from learning interdependent sets of feature weights. The use of the batch normalization as a second form of regularization, increases the stability of the model by normalizing the output of the previous layer, i.e., by subtracting the batch mean and dividing by the batch standard deviation. This layer has its own trainable parameters that are learned along with the remaining model parameters. Before computing the loss, the final embeddings are normalized to unit $L_{2}$ norm, which is a common strategy that proved beneficial in the open literature when training models with angular margin losses [34,35].

### 3.6. Discriminative Feature Learning Approach for Deep Palmprint Recognition

The training process for the proposed palmprint recognition model is done by using a combined learning objective of the following form:

(10) $$L=L_{aam}+\lambda L_{C},$$

where

(11) $$L_{aam}=-\frac{1}{M}\sum_{i=1}^{M}\log\frac{e^{s(\cos\left(\Theta_{y_{i}}+m\right)}}{e^{s(\cos\left(\Theta_{y_{i}}+m\right)}+\sum_{j=1,j\neq y_{i}}^{n}e^{s\cos\Theta_{y_{j}}}},$$

and

(12) $$L_{C}=\frac{1}{2}\sum_{i=1}^{M}\left|\right|x_{i}-c_{y_{i}}{\left|\right|}_{2}^{2}.$$

In the above equations, $L_{aam}$ is the Additive Angular Margin (aam) loss, also known as the ArcFace loss [34], $L_{C}$ is the center loss [36] and λ is a hyper parameter that balances the two [36]. In Equation (11), *m* denotes the angular margin penalty on angle $\Theta_{i}$, *s* is a feature scale parameter, $\cos\Theta_{y_{j}}$ is the logit for each class $y_{j}$, while the batch size and the class number are denoted as *M* and *n*, respectively. For the center loss in Equation (12), $x_{i}$ denotes the *i*-th feature vector belonging to the $y_{i}$-th class, and $c_{y_{i}}$ is the $y_{i}$-th class center. The center update in the center loss function is done over mini-batches as follows:

(13) $$c_{j}^{t+1}=c_{j}-\alpha \cdot \Delta c_{j}^{t},$$

where

(14) $$\Delta c_{j}^{t}=\frac{\sum_{i=1}^{M}\delta \left(y_{i}=j\right)\cdot \left(c_{i}=x_{i}\right)}{1+\sum_{i=1}^{M}\delta \left(y_{i}=j\right)}.$$

The hyper parameter α controls the learning rates of the centers and helps to avoid large perturbations caused by a few mislabelled samples, $\Delta c_{j}^{t}$ (Equation (14)) denotes the difference between the current center vector and the individual feature vectors, *t* stands for the iteration number, and δ(·) is equal to 1 when the inside condition is satisfied and 0 otherwise.

The motivation for using the combined loss is two-fold: (i) the ArcFace loss $L_{aam}$ contributes towards better separation between classes in the embedding space and ensures high discriminability of the learned feature representations, whereas (ii) the center loss $L_{C}$ minimizes intra-class variations and encourages features from the same classes to be pulled closer to their corresponding class centers [36,37]. While the ArcFace loss was originally designed to avoid the need for additional (discriminative) learning objectives (such as center loss), we show in the experimental section that the combination of the two losses still leads to improved performance.

### 3.7. Model Implementation

We implement our model in the PyTorch framework (available at: https://pytorch.org latest accessed on 10 November 2021) and use a built-in implementation of the VGG-16 model initialized with weights learned on the ImageNet dataset for all of our backbone feature extractors. We modify the backbone models in order to accept arbitrary sized input images by replacing the last max pooling layer in both processing paths with the average pooling layer with kernel size of 2, which yields 2048-dimensional embeddings. We set the number of patches in the local processing path to N=9 with a size of 75×75 pixels, which in our preliminary experiments was observed to provide a good trade-off between performance improvement of the overall model and its computational complexity. Both the global (holistic) and the aggregated local features are then concatenated to form a 4096 dimensional feature representation. After that, the combined feature vector is fed to a series of layers as summarized in Table 1 and illustrated in Figure 3d. Additional implementation details can be found in the publicly available source code: https://github.com/Marjan1111/tpa-cnn latest accessed on 10 November 2021.

Throughout all of our experiments we compare the global processing path (Figure 4) as a standalone CNN feature extractor against the two-path CNN feature extractor (Figure 5) in order to explore how the model benefits from using both global and local image information.

## 4. Experiments

### 4.1. Experimental Datasets

Two publicly available contactless palmprint datasets are used to evaluate the performance of the proposed method in this study, namely the IITD and CASIA palmprint datasets. Details on the two datasets are given below.

#### 4.1.1. IITD Palmprint Image Dataset

**Figure 7:**
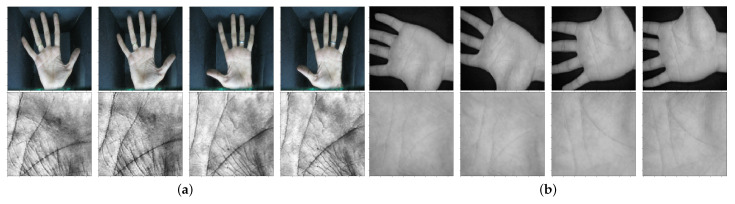
Samples from the left and right palmprints with their corresponding segmented region of interests (ROIs) for: (**a**) the IITD dataset, and for (**b**) the CASIA dataset.

The IIT Delhi (IITD) Touchless Palmprint Dataset [23], collected by the Indian Institute of Technology Delhi, provides images from the left and right hands of more than 230 volunteers in the age group between 14–57 years. Images in the database were collected with a simple touchless imaging setup in an indoor environment and with a circular fluorescent illumination around the camera lens. A total of 2601 color hand images from 460 palms with 7 samples for each hand of each person were acquired in a single session. The IITD database also provides automatically segmented (i.e., extracted Regions-Of-Interests—ROIs) and normalized images with the size of 150×150 pixels and at least 5 samples for each of the two hands. Some typical example images from the IITD dataset from the left and right palmprints with their corresponding ROIs are shown in Figure 7a.

#### 4.1.2. CASIA Palmprint Image Dataset

The second palmprint database used in our experiments is the CASIA Palmprint Image Dataset [24] (or CASIA-palmprint for short), collected by the Institute of Automation of the Chinese Academy of Sciences, which is one of the largest (to date) palmprint database available in terms of the number of individuals [24]. It contains 5052 palmprint images captured from 312 individuals, both males and females, with at least 8 samples for each hand of each individual acquired in a single session. The images were acquired by a self-developed palmprint recognition device with a CMOS imaging sensor fixed on top of it. No pegs were used to restrict postures and positions of the palms. The acquired images are in an 8-bit grayscale format with dimensions of 640×480 pixels. During data capture, the subjects were required to put their palms into the device and lay them on a uniform-colored background, to generate evenly distributed illumination. As explained in [2], there are several things that need to be considered w.r.t. this database. The individual “101” is the same as individual “19”, and therefore these two classes were merged into one class. The 11th image from the left hand of individual “270” was misplaced to the right hand, and the 3rd from the left hand of individual “76” represents a distorted sample with very poor quality. These problematic images were excluded from our experiments. As the CASIA database does not provide segmented palmprint images, we used the segmentation procedure from [6] to extract the palmprint ROIs. The final dataset adopted for our evaluation contained 301 individuals and a total of 5467 palmprint ROIs. A few illustrative sample images from the dataset are presented in Figure 7b.

### 4.2. Experimental Setup

To evaluate the performance of the proposed model, verification experiments were conducted over the two experimental datasets. Every two samples in the datasets are matched. If the two images were from one person (class), the matching is considered genuine (a mated comparison), otherwise, it was considered an impostor matching (non-mated comparison). Following the all-vs.-all experimental protocol, the number of genuine verification attempts conducted ($N_{genuine}$) and the number of impostor attempts ($N_{impostor}$) can be computed with Equations (15) and (16), as follows:

(15) $$N_{genuine}=N_{hands}\times N_{enroll}\times N_{class},$$

(16) $$N_{impostor}=N_{hands}\times N_{enroll}\times N_{class}\times \left(N_{class}-1\right),$$

where $N_{hands}$ is the number of hands, $N_{enroll}$ is the number of enrollment palmprint images of the the subjects, and $N_{class}$ is the number of unique classes in a given dataset. The cosine similarity function is used to compute client and impostor matching scores *S*, as seen in Equation (17), where $n^{\prime }$ is the length of the feature vector, $f_{p}$ is the feature vector extracted from the given probe image, and $f_{e}$ is the feature vector extracted from the enrollment image.

(17) $$S=\frac{f_{p}\cdot f_{e}}{∥\;f_{p}∥∥\;f_{e}∥}=\frac{\sum_{i=1}^{n^{\prime }}f_{p,i}f_{e,i}}{\sqrt{\sum_{i=1}^{n^{\prime }}f_{p,i}^{2}}\cdot \sqrt{\sum_{i=1}^{n^{\prime }}f_{e,i}^{2}}},$$

where · denotes the scalar product, ||·|| is the $L_{2}$ norm operator, and $f_{\cdot ,i}$ is the *i*-th element of vector $f_{\cdot }$.

For the experiments we partition the image datasets into three disjoint subsets, i.e., (i) a training set, which is used to learn the model parameters, (ii) a validation set, a subset of the overall training data, used to monitor the training progress and provide an unbiased estimate of the model fit when tuning hyperparameters, and (iii) a testing set which is utilized to evaluate the final verification performance.

Since the two datasets contain different numbers of samples per class, we perform a simple undersampling process. This process ensures that each class has as many samples as the class with the minimum number of samples. The number of samples in the datasets before and after the undersampling are shown in Table 2. By using this process, we balance the datasets with the goal of removing potential biases for the training procedure. On the other hand, the undersampling discards some potentially valuable sources of information. We address this issue through severe data augmentations that further stimulate training and prevent overfitting.

### 4.3. Performance Metrics

Following standard methodology [32,38,39], the following error rates commonly used for verification experiments are adopted to evaluate the performance of the tested models:

- **Area Under the Curve (AUC):** AUC represents a performance metric that measures the overall performance of a learned binary model and is typically computed from a standard Receiver Operating Characteristic (ROC) curve. This metric is widely used to assess performance of biometric systems operating in verification mode. A poor model fit results in AUC ≈0.5 which indicates randomness, on the other hand, a good model fit results in AUC ≈1.
- **Verification Rate at the False Accept Rate of 0.1% (VER@0.1FAR):** This performance measure corresponds to an operating point on the ROC curve and is computed as follows:
  (18) $$\mathrm{VER}@0.1\mathrm{FAR}=1-\mathrm{FRR}(\theta_{ver01}),\mathrm{where}$$
  (19) $$\theta_{ver01}=\arg\min_{\theta }\left|\mathrm{FAR}\left(\theta \right)-0.001\right|,$$
  where $\theta_{ver01}$ is the relevant decision threshold, FAR denotes the False Accept Rate, and FRR is the False Reject Rate. FAR corresponds to the percentage/fraction of times an invalid (impostor) user is accepted by the system and is calculated as follows:
  (20) $$\mathrm{FAR}\left(\theta_{k}\right)=\frac{\left\{|d_{imp}|d_{imp}\leq \theta_{k}|\right\}}{\left\{|d_{imp}|\right\}}.$$
  Similarly, FRR represents the percentage/fraction of times a valid (genuine) user is rejected by the system and is calculated as follows:
  (21) $$\mathrm{FRR}\left(\theta_{k}\right)=\frac{\left\{|d_{gen}|d_{gen}\geq \theta_{k}|\right\}}{\left\{|d_{gen}|\right\}}.$$
  In Equations (20) and (21), $\left\{d_{imp}\right\}$ and $\left\{d_{gen}\right\}$ represent sets of impostor and genuine scores generated during the experiments, respectively, |·| denotes a cardinality measure, and $\theta_{k}$ represents the decision threshold.
- **Verification Rate at the False Accept Rate of 1% (VER@1FAR):** This measure corresponds to another operating point on the ROC curve defined as follows:
  (22) $$\mathrm{VER}@1\mathrm{FAR}=1-\mathrm{FRR}(\theta_{ver1}),\mathrm{where}$$
  (23) $$\theta_{ver1}=\arg\min_{\theta }\left|\mathrm{FAR}\left(\theta \right)-0.01\right|,$$
  where $\theta_{ver1}$ denotes the relevant decision threshold.
- **Equal Error Rate (EER):** The last performance measure, EER, is defined with a decision threshold $\theta_{eer}$ that ensures equal values of FAR and FRR as follows:
  (24) $$\mathrm{EER}=\frac{1}{2}\left(\mathrm{FAR}\left(\theta_{eer}\right)+\mathrm{FRR}\left(\theta_{eer}\right)\right),\mathrm{with}$$
  (25) $$\theta_{eer}=\arg\min_{\theta }\left|\mathrm{FAR}\left(\theta \right)-\mathrm{FRR}\left(\theta \right)\right|.$$

Additionally, we also use Receiver Operating Characteristic (ROC) curves to visualize the performance of the models throughout all experiments. Note that all performance scores defined above are provided in % when reporting results.

### 4.4. Training Details

We train our model(s) with the combined objective function from Equation (10). During the training process, some hyperparameters remain fixed, while others are further analyzed in a broader range to study their impact. The parameters that we vary in our experiments are the hyper-parameters of the combined objective function, i.e., the λ parameter, which balances the impact of the ArcFace and the center losses, and the parameter α that controls the learning rate of the centers in the center loss function. The optimization of the base model parameters is done with the Adam optimizer with a learning rate of $1\times 10^{-4}$. The channel-wise attention-pooling operation, which has its own trainable parameters, is trained with a learning rate of $1\times 10^{-2}$.

To prevent overfitting we use severe data augmentations and modify data on the fly, so that our CNN models is transformation invariant to the maximum possible extent. The augmentations are done with the Imgaug (available at: https://github.com/aleju/imgaug, accessed on 10 November 2021) library and the following image transformations:

- Histogram equalization,
- Rotations in the range ±10°,
- Random cropping (with a 50% chance), where between 5% and 30% of the original image is cropped away,
- Multiplication of all image pixels with a random value *v* sampled once per image in the range (0.9, 1.2),
- Application of one of the following:
  (1) Random corruption of *p* percent of all pixels within an image area by salt-and-pepper noise, where p∈(1,10)%. The size of the image area is between 2% and 20% of the size of the input image,
  (2) Random replacement of a certain fraction of pixels from the image with zero, with a percentage value p∈(1,10)%, with 20% per-channel probability,
  (3) Random replacement of rectangular masks from the image with zeros, with a percentage value p∈(1,10)%. The size of the masks is between 2% and 20% of the size of the input image,
- Application of one of the following:
  (1) Blurring with a Gaussian kernel with a sigma value between (0,3.0),
  (2) Blurring using averaging over neighborhoods that have random sizes, which can vary between 5 and 11 in height and 1 and 3 in width,
  (3) Application of motion blur with a kernel size of 15×15 pixels.

Through these data augmentations we ensure that our model sees new variations of the data at each and every epoch of the training procedure, and (almost) never sees the exact same image multiple times, which is always beneficial when fine-tuning models trained initially on large scale datasets like ImageNet to downstream tasks. By carefully choosing the image augmentations, we can additionally boost the predictive performance of the learned model, which further enhances its generalization capability. Another reason to use data augmentation is because we want to lower the gap between the training and validation performance during the training process. All augmentations are performed in random order and with 50% probability, which means there is a (small) chance for not performing augmentations at all.

**Figure 8:**
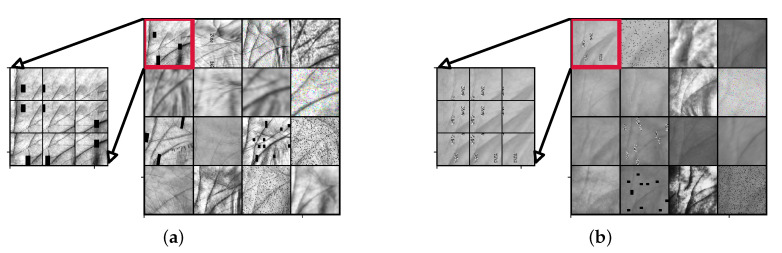
Illustration of different data augmentations as well as the patch decomposition procedure for sample images from (**a**) the IITD dataset and (**b**) the CASIA dataset.

For illustrative purposes, several image augmentations with the patch decomposition procedure from a single augmented image are shown in Figure 8a for IITD, and in Figure 8b for the CASIA dataset. It is important to note that the second input in the proposed Two-Path CNN model (Figure 5), takes the same augmented image as input, but decomposes it into *N* smaller patches sampled in a grid-like fashion with overlap. By doing this, we ensure that the global and the local processing path are looking at the same input data during training.

## 5. Quantitative Results

The main goal of this section is to rigorously evaluate the proposed Two-Path CNN palmprint recognition model and to compare its performance with competing state-of-the-art methods from the literature. Throughout the experiments, we evaluate all models on different subsets of images by randomly splitting the test data into five equal folds and adopting the all-vs-all experimental protocol in each of the folds. We use this approach to be able to also estimate confidence scores on all reported results in addition to the overall performance.

For the competing methods we report results for several traditional feature extraction methods, using the code provided in [37,40]. Furthermore, we also compare our results against four state-of-the-art methods from the literature, i.e., three DL-based from [4,31,41], and one traditional (non DL-based) solution [11], previously reported to achieve state-of-the-art performance on standard benchmarks.

### 5.1. Comparison with Competing Methods

For the comparison with traditional feature extraction approaches we consider seven dense-descriptor based methods, i.e., Local Binary Patterns (LBPs) [42], Rotation Invariant and Local Phase Quantization (RILPQ [43] and LPQ [44]) features, Histograms of Oriented Gradients (HOG) [8,45], Binarized Statistical Image Features (BSIF) [46], Patterns of Oriented Edge Magnitudes (POEM) [47] and features extracted with the help of a bank of the Gabor filters [48]. These feature extraction techniques are used (with default parameters from [40]) to infer compact representations from the palmprint images for matching. To facilitate a fair comparison, the cosine similarity is again used to produce the comparison scores.

**Figure 9:**
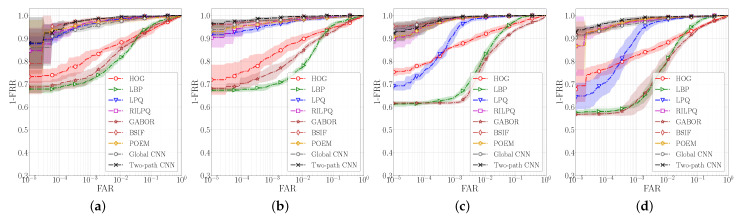
Comparison between the traditional feature extraction methods and the proposed two-path model evaluated on the (**a**) left and (**b**) right palmprints from the IITS dataaset, as well as the (**c**) left and (**d**) right palmprints of the CASIA dataset. The figure shows average ROC curves together with confidence intervals (in the form μ±σ) on a semilog scale.

The results of the comparison are presented in the form of ROC curves in Figure 9 and in the form of performance scores in Table 3. We can see that the proposed Two-path CNN approach clearly outperforms all traditional feature extraction methods at every evaluated operating point, with BSIF, RILPQ and POEM being the closest competitors. We also observe that the Global-CNN, when used as a standalone feature extractor, is able to outperform the traditional methods in almost all cases except for BSIF, RILPQ and POEM, when evaluated on the right palmprints from the CASIA dataset. Additionally, we notice that the local image properties captured by recognition techniques based on HOG, LBP and Gabor features, are not sufficient to extract useful information from contactless palmprint images where the presence of elastic deformations is known to be problematic. We can conclude that even though the Global-CNN model processes the input image in a holistic manner, the very nature of the CNN models helps to extract discriminative local information due to the local connectivity of the convolutional kernels. However, the addition of the local image properties through the local processing path in the proposed Two-path model boosts the verification performance while making the deeply learned features more discriminative and more resilient to the presence of elastic deformations and other artefacts caused by the contactless acquisition procedure.

### 5.2. Comparison with State-of-the-Art Methods

Next, we compare the proposed method against three state-of-the-art DL-based palmprint recognition techniques and one non DL-based method. To ensure a fair comparison, we either use the evaluation protocol used by the authors of the baseline techniques or utilize the code provided by the authors of the baseline methods (where available), and use our evaluation protocol for the comparison.

The first state-of-the-art method is a highly optimized DL architecture, referred to as Residual Feature Network (RFN) [4]. RFN is based on a fully convolutional network that generates deeply learned residual features trained with the Soft-Shifted Triplet Loss (SSTL). The key advantage of this method is that it offers superior generalization capabilities across different datasets. FRN-SSTL does not have fully connected layers which results in pure feature map outputs and is beneficial for preserving spatial correspondences among the most discriminative palmprint areas. The second method is referred to as Difference of Vertex Normal Vectors (DoN) and represents a non-DL based method [11]. The key advantage of DoN lies in the ability to extract 3D information from 2D palmprint images, which is beneficial when dealing with contactless palmprint images, where the presence of different variations in illuminations is common. The next method selected for the comparison represents a DL approach initially presented in [31] and uses the GoogLeNet DL architecture as a backbone feature extractor. We denote this method as AML_N-Pair in this paper. To obtain discriminative and equidistributed embeddings, the authors propose a novel optimization objective which consists of a pair of adversarial loss functions, specifically, a distance metric term and a confusion term. By using the joint supervision of those two terms, this method was reported to achieve strong generalization in cross-dataset contactless palmprint recognition experiments. We also compare the results against the Weight-based Meta Metric Learning (W2ML) approach proposed in [41]. This method uses metric learning in a meta way to extract discriminative palmprint features using an end-to-end DL network, which was shown to be robust and efficient for diverse palmprint recognition scenarios.

It is important to note that the experimental results are done only on the IITD dataset since some authors did not provide results for the CASIA dataset. Table 4 summarizes the experimental results for IITD in terms of EER values. The proposed Two-path model clearly outperforms the DoN, AML_N-Pair and W2ML approaches and ensures an EER reduction of around 50% compared to any of these methods. Additionally, it performs comparably to the recent RFN-SSTL method, where a very similar EER score is achieved by the proposed approach. The difference in the point estimates of the EER scores of RFN-SSTL and our approach is statistically not significant. The reported results point to the discriminative power of feature representations generated by the proposed Two-path model and their suitability of contactless palmprint recognition. Furthermore, we note that we are able to further improve on the results reported here by explicitly modeling elastic deformations using a Thin Plate Spline (TPS) transformation. We provide detailed information on this process (not yet used for generating results in this section) in Section 5.4.

### 5.3. Sensitivity Analysis and Ablations

In this section for the ablation study we conduct a wide range of experiments by examining different parts of our method.

To explore the impact of various model components on performance we conduct a sensitivity analysis. Specifically, we investigate the sensitiveness of models to changes of the parameters in the combined training objective, i.e., the λ parameter (Equation (10)), which balance the contribution of the two learning objectives, and the α parameter that controls the learning rate of the centers (Equation (13)) in the center loss function. Note that the experiments also include cases where λ=0 and, hence, one of the loss terms is ablated. Additionally, we also provide a detailed look into the performance of the global and local processing paths and their contributions to the overall performance of the complete Two-path model.

#### 5.3.1. Impact of λ and α Parameters

In the next series of experiments we train multiple models with different λ and α parameters and investigate how sensitive the training procedure is to changes in hyper-parameter values and how these values affect performance. Note that we use only the IITD dataset for this sensitivity analysis. In the first experiment, we fix the α parameter to 0.5 and vary λ from 0 to 0.1, and in the second experiment we vary α between 0.2 and 1, while using the value of λ from the best performing model obtained in the first experiment. The reason why we restrict α in that specific range is because when using values below 0.2, we observed that the model did not converge.

**Figure 10:**
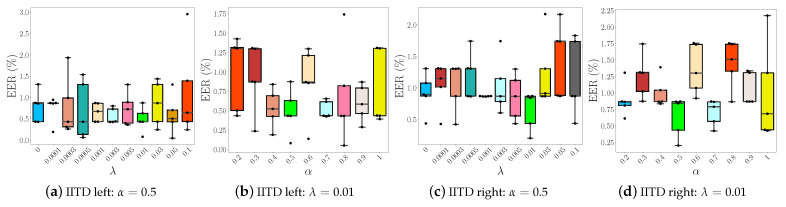
Results of the sensitivity analysis in the form of box plots of the generated EER scores. The Two-path CNN model is trained with different combinations of λ and α parameters on: (**a**) IITD left, (**b**) IITD right, (**c**) CASIA left and (**d**) CASIA right palmprints, respectively. The figure is best viewed in color.

The results of the analysis are reported in the form of box plots of the calculated EER scores in Figure 10. To highlight the differences between the learned models we use different colors for each box plot, where the best and the worst performing ones are highlighted with lime and red colors, respectively. We can clearly see that the best performing model when trained on IITD left palmprints (Figure 10a,b), is obtained when λ=0.01 and α=0.5. The same applies when the model is trained on the right IITD palmprints. We notice that the model trained only with the ArcFace loss already ensures competitive performance. However, the inclusion of the center loss with the right set of parameters is observed to further improve results. Thus, the box-plots clearly show that both loss terms are important for the proposed model.

#### 5.3.2. Contribution of Global and Local Processing Paths

The proposed Two-path model consist of two distinct processing paths. To explore the contribution of the two paths to the overall performance of the proposed model, we extract the global features as well as the local features before the concatenation operation and perform verification experiments based on each image representation. We compare the performance of the two processing paths with the full two-path model in Table 5. It is evident that the use of combined image information plays an important role to further improve the verification performance of the global processing path. The addition of local information consistently improves results and allows the Two-path model to produce more reliable verification decisions. In terms of EER, for example, we observe relative error reductions ranging from 6% and up to 48%—depending on the dataset and the used experimental setup. Thus, the information added by the local processing path clearly complements the information extracted by the global path and helps addressing issues caused by the contactless acquisition procedure. When interpreting the reported results, it is important to note that the complete two-path model is trained end-to-end, so the performance of the learned global and local representations needs to be considered in this context. The reported performances for the global and local features are, therefore, correctly interpreted as the partial contributions of the processing paths to the overall performance of the model and not in terms of capacities of the individual representations for recognition. The performances here would be quite different if the representations would be trained one without the other.

### 5.4. Explicit Modeling of Elastic Deformations

In this series of experiments we try to explicitly address the problem of elastic deformations which represent one of the major problems with contactless palmprint recognition techniques.

A common way to tackle this problem is to achieve invariance to warping through some kind of spatial transformation on the input images. Based on this insight, we use a Spatial Transformer Network (STN) [49,50] to learn a warping procedure based on Thin Plate Splines (TPS). TPS is selected as the basis for warping because it ensures a higher degree of freedom when modeling deformations compared to other affine transformations. The TPS algorithm has been applied successfully in several computer vision applications, especially in medical image registration [51], as well as a minutiae matching algorithms that model the elastic deformations in fingerprint images [52]. For detailed explanation about the STN and TPS, the reader is referred to [49,50,53], respectively. However, to the best of our knowledge, it has not yet been applied as a warping technique for modelling elastic deformations in palmprint images acquired in a contactless manner. It is important to note that we did not integrate the STN directly into our Two-path model. Instead we used it as an external source to generate TPS warped images. This means that in each iteration we generate an additional batch of warped images which are then combined with the current batch of images when training the model. With this procedure, we are forcing our augmentations to be TPS warped with 50% chance.

**Figure 11:**
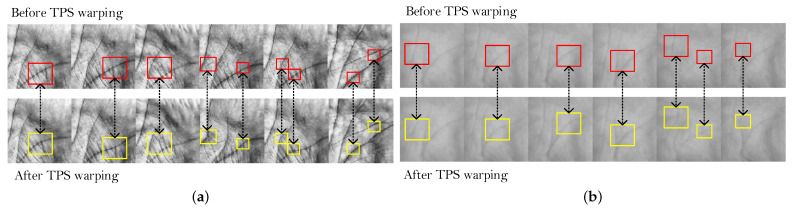
Visual effect of the TPS-based modeling of elastic deformations. Examples are shown before and after the TPS warping on sample images from: (**a**) the IITD dataset and (**b**) the CASIA dataset. The biggest differences after TPS warping are seen in the principle lines. The regions where the principle lines are affected the most by the warping process are marked with rectangles.

**Figure 12:**
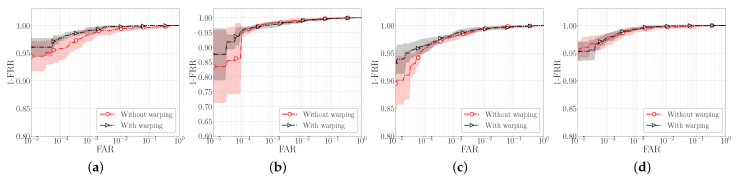
Impact of using TPS warping as an augmentation technique on the verification performance. The results are shown in the form of mean ROC curves with confidence intervals when the models are trained on: (**a**) IITD left, (**b**) IITD right, (**c**) CASIA left and (**d**) CASIA right palmprints, respectively.

**Figure 13:**
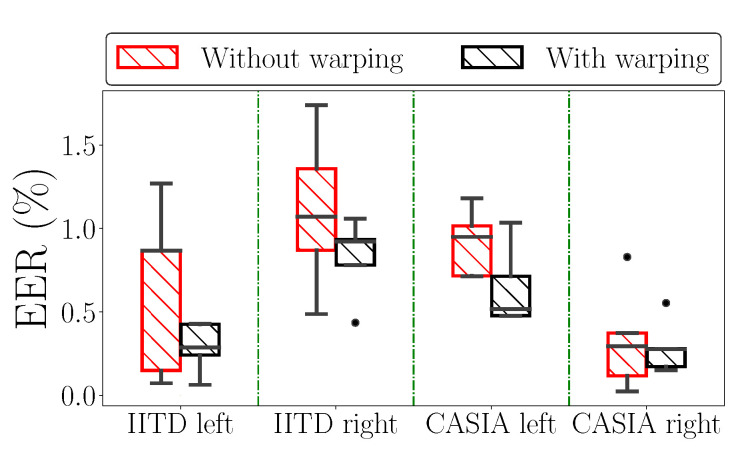
Equal error rate (EER) box plot visualization showing the effect before and after including the TPS warping as image transformation in the existing augmentations when the Two-path architecture is trained on either left or right palmprints from IITD and CASIA datasets.

The results before and after the TPS-STN warping applied on sample images from the IITD and CASIA datasets are shown in Figure 11. We can clearly see that the images after the warping procedure are somewhat deformed. This effect can be mostly seen around the principal lines of the palmprint, which is also the region where elastic deformations are most prominent. The ROC curves generated with and without data augmentation based on TPS warping are shown in Figure 12, and the respective box-plots of the corresponding EER scores are presented in Figure 13. As can be seen from the results, the TPS warping has a significant impact on the verification performance, which results in lower EER values and narrower confidence intervals.

## 6. Qualitative Results

In this section we present different qualitative results related to the Two-path architecture. We mainly focus on the following analyses:

- Exploring the discriminative power of the deeply learned embeddings through the T-distributed Stochastic Neighbor Embedding (t-SNE) [54] data visualization technique,
- Investigating the feature importance and attention mechanism of the Two-path architecture,
- Qualitative evaluations of edge cases generated in the verification experiments.

### 6.1. Embedding Visualization with t-SNE

We use t-Distributed Stochastic Neighbor Embedding (t-SNE) [54] to visualize the high dimensional embeddings generated by the proposed model from IITD and CASIA images. t-SNE represents a nonlinear dimensionality reduction technique developed by the authors in [54], which is suitable for embedding high-dimensional data for visualization into a low-dimensional space of two or three dimensions. For a detailed explanation about the t-SNE technique, the reader is referred to [54].

**Figure 14:**
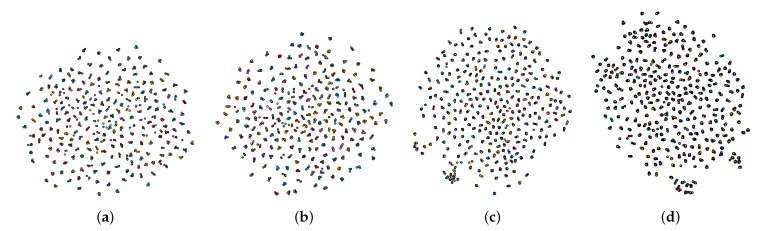
2-D t-SNE visualization of the learned palmprint embeddings for sample images from the (**a**) IITD left, (**b**) IITD right, (**c**) CASIA left, and (**d**) CASIA right palmprints. Note that the combination of the learning objectives results in well separated and compact class clusters. The figure is best viewed electronically and zoomed-in.

In our case, we use t-SNE to generate 2-D visualizations in order to see the clustering effect produced from the joint supervision between ArcFace loss and the center loss. In Figure 14 the learned 2-D embeddings for IITD and CASIA datasets are shown. From the 2-D representation, we can clearly see the effect of the center loss which efficiently pulls the features to their corresponding centers, thus, minimizing the intra-class variations. On the other hand, the ArcFace loss helps the embeddings from different classes to spread uniformly on the hyperspace by maximizing the inter-class variations. We also notice that the embeddings from the CASIA dataset are less uniformly spread compared to the embeddings from IITD dataset. This effect can be seen from Figure 14c, where we can see regions of less discriminative embeddings. As seen from Figure 14d, there are also regions where the discriminative power between the embeddings is preserved, however these regions suffer from high locality, i.e., there are features located in several small areas of the embedding space isolated from the rest of the embeddings. Such cases where the combined objective did not produce sufficiently discriminative features can possibly lead to miss-classifications of palmprint images.

### 6.2. Feature Importance and the Attention Mechanism

**Figure 15:**
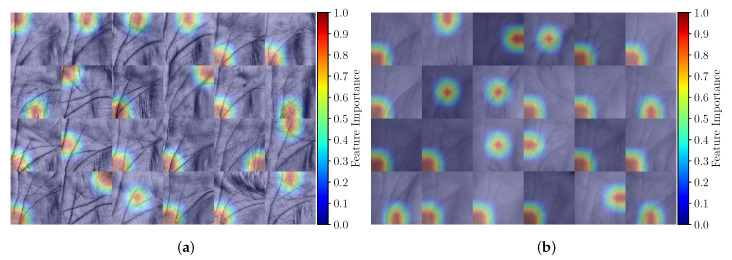
Exploring the feature importance learned from the attention mechanism for the: (**a**) IITD dataset and (**b**) CASIA dataset.

Next, we report qualitative results with respect to the attention mechanism integrated within the proposed Two-path architecture. As discussed earlier (in Section 3.4), the so called channel-wise attention pooling mechanism is a trainable attention operation which offers a straight-forward way of exploring the importance of each image part through the learned feature vector of weights. This feature vector is then converted to a vector of probabilities by using the softmax activation function. To generate interpretable results, we transform the learned vector of probabilities into heatmaps and then superimpose the computed heatmaps over the palmprint images. In Figure 15a,b some qualitative evaluations for the IITD and CASIA datasets are shown, respectively. We can see how the attention mechanism selects certain image regions that are not only locally important but are also beneficial for the overall image representation. Note how this procedure is adaptive in nature and selects a different area in each palmprint depending on image characteristics. Additionally, the selected areas are also quite localized due to the use of the softmax function in the attention mechanism.

### 6.3. Verification Experiment

Finally, we present qualitative results related to the verification experiments conducted on the IITS and CASIA dataset. Specifically, we are interested in separability of the score distributions for the mated and non-mated experiments as well as failure cases generated by our Two-path model.

**Figure 16:**
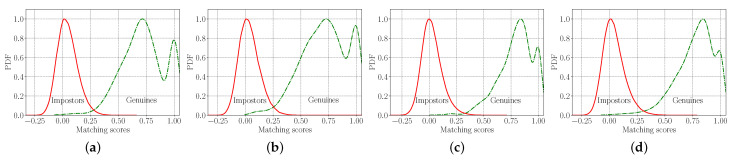
Genuine-impostor score distributions expressed as probability density functions (PDFs) when evaluating on different palmprints: (**a**) IITD left palmprints, (**b**) IITD right palmprints, (**c**) CASIA left palmprints and (**d**) CASIA right palmprints.

In Figure 16 the mated and non-mated score distributions expressed as probability density functions (PDFs) [1] when evaluating on different palmprints from the datasets are shown. It can be observed that the Two-path architecture achieved good separation between the genuine and impostors score distributions, where the combined learning objective efficiently minimized the intra-class variations, while maximizing the inter-class variations, which validates our previous analyses. It is also clear that the impostor score distributions are centered at zero, which points to the fact that inter-class embeddings are well distributed in the hyper feature space [55]. Here we can also see the presence of the “double-peak” phenomenon [55]—in our case in the mated (genuine) score distributions [55]. This implies that some features of same classes exhibit weaker similarities and are therefore harder to recognize.

Next, we preform an verification experiment based on the decision threshold $\theta_{eer}$, which ensures equal values of the FAR and FRR. Since there is a slight overlap between the score distributions, we can expect that some of the genuine verification attempts will be falsely rejected, i.e., false negatives (FN), and vice versa, some of the impostor verification attempts will be falsely accepted i.e., false positives (FP). For illustration purposes, we perform the experiment on the palmprint images from both datasets. We present the results in Table 6 by visualizing some of the compared image pairs and their respective cosine similarity scores.

As can be seen from Table 6, the matching scores between the correctly accepted mated/genuine attempts, i.e., true positives (TP), are positive and above the decision threshold, whereas the matching scores between the correctly rejected impostor attempts, i.e., true negatives (TN), are negative, which indicates opposite feature vectors. The results in the overlapping region between the genuine and impostor distribution are also quite interesting. As seen from the falsely rejected genuine attempts or FNs, palmprints from the same subjects share less similarities due to the high appearance variability caused by the palmprint acquisition process. For example, in CASIA right palmprint images there is an extreme case where one palmprint image between the genuine matches is completely white due to high illuminations that happened during the palmprint acquisition process. This means that the Two-path architecture is not able to find any shared patterns between such images, which results in lower matching score. On the other hand, there are cases where the exact opposite happens. Different palmprints from different individuals share more similarities, which results in relatively high scores and above the threshold similarity scores.

## 7. Conclusions

In this study we introduced a hybrid Two-path DL based model for contactless palmprint recognition. Unlike competing hand-crafted feature extraction techniques, our architecture automatically learns discriminative features from the data while not requiring extensive parameters tuning. Compared to other DL techniques, our model is designed as dual path CNN, where one input encodes the input image holistically using a backbone CNN-based feature extractor, whereas the second path, a parallel Siamese architecture with shared model parameters, captures local information from image patches sampled from the input image. Moreover, we showed that the discriminative power of the deeply learned features can be highly enhanced by combining the ArcFace loss with the center loss to jointly supervise the learning of our model. By using this design we were able to achieve great genuine-impostor score distribution separation which is key in biometric recognition systems and paramount for achieving state-of-the-art performance on standard benchmarks.

We also performed extensive experiments related to different parts of the architecture and showed how the addition of local information contributes to enhanced robustness to elastic deformations, as well as, increased generalization capability across different datasets. Furthermore, we showed that our design choices ensure a level of explainability facilitated by the proposed channel-wise attention pooling mechanism. Given the promising results, further extension of this work will focus on palmprint recognition in the wild and explore how the model generalizes when images are acquired under complex backgrounds, in different environments and with various postures and illuminations. Furthermore, different feature pooling strategies and attention mechanisms that could maximize the information content that can be extracted from the local image patches, especially when considering detailed image aligned will also be investigated. Particular attention will be payed to the impact of alignment on the patch generation strategy used.

## Figures and Tables

**Table 1:** Description of the used layers with their corresponding output dimensions after the concatenation procedure. This part of the model is shown in Figure 3d.

Layer Abbreviation	Type of Layer	Output Dimensions
$f_{c}$	Input	4096
FC	Fully Connected	4096
Bn	Batch Normalization	2048
ReLU	Activation Function	2048
FC	Fully Connected	2048
Bn	Batch Normalization	1024
$L_{2}$	$L_{2}$ Normalization	1024

**Table 2:** The amount of data before and after the application of the undersampling procedure on the two experimental datasets.

Dataset	Palm	
	Left	Right
IITD (min. samples per class)	min = 5	min = 5
Initial numbers	1300	1301
After undersampling	1150	1150
CASIA (min. samples per class)	min = 6	min = 7
Initial numbers	2728	2739
After undersampling	1806	2107

**Table 3:** Comparison against traditional recognition approaches relying on hand-crafted features. Results are reported for left and right palms separately for both datasets. The results are presented in the form of a μ±σ (and in (%)) computed over five test subsets. The best performance is highlighted in bold.

Method	HOG [45]	LBP [42]	LPQ [44]	RILPQ [43]	Gabor [48]	BSIF [46]	POEM [47]	Global (Ours)	Two-Path (Ours)
	**Evaluated on the Left Palmprints from IITD Dataset**								
EER	7.216±1.519	6.380±0.577	1.383±0.578	1.824±0.508	7.123±0.744	1.121±0.431	1.737±1.014	1.849±0.641	0.757±0.212
AUC	97.394±0.690	98.072±0.437	99.812±0.133	99.660±0.289	97.495±0.694	99.768±0.142	99.359±0.442	99.770±0.127	99.935±0.065
VER@0.1FAR	81.739±3.049	71.652±1.272	96.173±1.006	97.130±0.650	73.739±2.730	98.0±0.976	96.434±1.516	94.782±1.099	98.086±0.347
VER@1FAR	88.086±2.104	81.391±0.968	98.521±0.706	98.0±0.650	85.304±1.659	98.869±0.650	97.913±1.358	97.478±1.179	99.478±0.507
	**Evaluated on the Right Palmprints from IITD Dataset**								
EER	6.327±1.041	7.209±0.488	1.309±0.266	1.101±0.428	6.184±0.866	1.191±0.453	1.652±0.426	0.900±0.421	0.602±0.234
AUC	97.796±0.691	97.607±0.496	99.868±0.090	99.855±0.123	98.050±0.335	99.780±0.121	99.701±0.191	99.939±0.056	99.912±0.102
VER@0.1FAR	82.434±0.706	69.304±1.307	96.434±0.886	97.565±0.706	75.217±1.760	97.565±0.706	96.695±0.806	97.391±1.626	98.521±0.589
VER@1FAR	88.608±0.325	77.043±2.599	98.608±0.325	99.043±0.748	87.217±2.994	98.521±0.758	98.260±0.476	99.043±0.576	99.478±0.425
	**Evaluated on the Left Palmprints from CASIA Dataset**								
EER	5.025±1.005	5.150±0.663	0.904±0.336	0.705±0.216	7.530±0.475	0.465±0.230	0.679±0.117	0.385±0.273	0.374±0.164
AUC	98.557±0.546	98.992±0.174	99.896±0.095	99.947±0.060	96.704±0.418	99.969±0.039	99.939±0.046	99.987±0.011	99.986±0.015
VER@0.1FAR	86.489±0.657	64.229±0.781	92.411±2.863	98.671±0.563	61.960±0.756	99.003±0.646	98.172±0.622	99.113±0.847	99.113±0.687
VER@1FAR	91.861±1.625	82.889±2.163	98.836±0.847	99.501±0.322	80.011±2.228	99.723±0.303	99.501±0.207	99.722±0.303	99.778±0.207
	**Evaluated on the Right Palmprints from CASIA Dataset**								
EER	6.691±0.691	5.577±0.618	1.533±0.421	0.949±0.336	7.677±0.334	0.981±0.487	0.997±0.275	1.208±0.306	0.553±0.315
AUC	97.756±0.234	98.725±0.256	99.659±0.030	99.885±0.064	97.065±0.471	99.770±0.225	99.695±0.167	99.801±0.089	99.917±0.070
VER@0.1FAR	82.631±1.561	61.652±1.595	90.792±4.662	97.436±0.317	62.938±5.086	97.864±1.093	97.959±0.465	96.583±0.906	98.625±0.863
VER@1FAR	88.041±1.516	80.305±1.449	98.101±0.721	98.907±0.512	79.879±3.645	99.051±0.541	99.003±0.274	98.718±0.439	99.525±0.299

**Table 4:** Comparison against state-of-the-art methods from the literature. The comparison is done on the IITD dataset in terms of EER values. All results are presented in (%).

Setting	Method	EER (in %)
Protocol from [4,11,41]	DoN [11]	1.391
	RFN-SSTL [4]	0.600
	W2ML [41]	2.330
	Two-Path CNN (ours)	0.701
Code from [31]	AML_N-Pair [31]	1.690
	Two-Path CNN (ours)	0.910

**Table 5:** Summary of averaged performance metrics when using different types of learned image descriptors. The results are presented in the form of a μ±σ and in (%).

Type of Descriptor	Global	Local	Two-Path (Combined)
	**Evaluated on the Left Palmprints from IITD Dataset**		
EER	1.1270±0.212	10.677±0.573	1.061±0.416
AUC	99.854±0.083	95.570±0.377	99.879±0.063
VER@0.1FAR	96.956±1.133	75.130±1.439	96.956±1.133
VER@1FAR	98.035±0.325	80.173±1.185	98.869±0.851
	**Evaluated on the Right Palmprints from IITD Dataset**		
EER	0.894±0.236	15.370±2.012	0.462±0.050
AUC	99.887±0.099	92.181±1.527	99.969±0.137
VER@01FAR	97.826±0.991	69.043±0.902	98.521±0.589
VER@1FAR	99.130±0.274	72.086±0.411	99.652±0.173
	**Evaluated on the Left Palmprints from CASIA Dataset**		
EER	0.413±0.296	18.310±0.690	0.331±0.111
AUC	99.959±0.040	91.465±0.334	99.982±0.006
VER@0.1FAR	99.114±0.687	62.568±0.776	99.335±0.414
VER@1FAR	99.723±0.349	65.115±0.555	99.778±0.207
	**Evaluated on the Right Palmprints from CASIA Dataset**		
EER	0.930±0.399	12.483±1.466	0.643±0.214
AUC	99.861±0.112	94.527±0.976	99.900±0.064
VER@0.1FAR	98.434±0.510	63.549±1.489	98.481±0.282
VER@1FAR	99.193±0.440	72.471±1.422	99.431±0.320

**Table 6:** Verification results based on the decision threshold $\theta_{eer}$ which ensures equal values of FAR and FRR. Image pairs are presented that generated different outcomes in the verification process, i.e., TP-true positives, FN-false negatives, FP-false positives, and TN-true negatives.

TP	FN	FP	TN	TP	FN	FP	TN
**IITD right palmprints, $\theta_{eer}=0.276$**				**CASIA right palmprints, $\theta_{eer}=0.295$**			
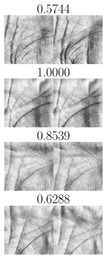	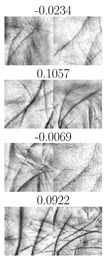	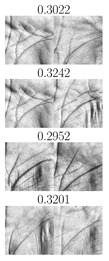	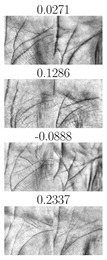	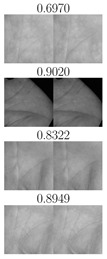	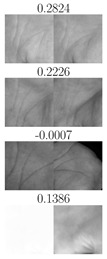	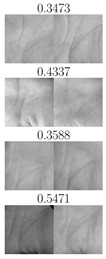	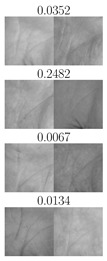
**IITD left palmprints, $\theta_{eer}=0.259$**				**CASIA left palmprints, $\theta_{eer}=0.307$**			
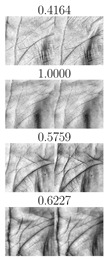	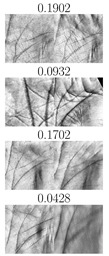	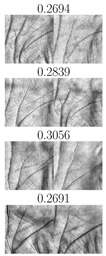	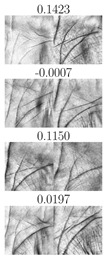	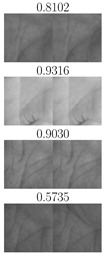	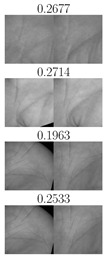	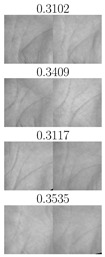	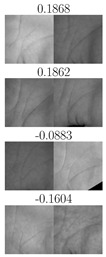

## Data Availability

The source code and models used in this paper are publicly available from GitHub: https://github.com/Marjan1111/tpa-cnn latest accessed on 10 November 2021.
